# Programming Effects of Maternal Nutrition on Intestinal Development and Microorganisms of Offspring: A Review on Pigs

**DOI:** 10.3390/microorganisms13051151

**Published:** 2025-05-17

**Authors:** Liang Hu, Fali Wu, Lianqiang Che

**Affiliations:** 1 Key Laboratory for Animal Disease-Resistant Nutrition of the Ministry of Education of China, Institute of Animal Nutrition, Sichuan Agricultural University, Chengdu 611130, China; wufali8848@163.com; 2 College of Food Science, Sichuan Agricultural University, Ya’an 625014, China

**Keywords:** intestine, immunity, microbiota, maternal nutrition, fetus

## Abstract

Intestinal development is a critical determinant of growth and overall health in pigs. Accumulating evidence underscores the significant influence of intestinal microbiota on essential physiological functions and systemic health. Dietary nutrients play a pivotal role in regulating both intestinal development and the composition of intestinal microbiota. Optimal early-life nutrient provision ensures proper intestinal growth and functional maturation, with maternal nutrition emerging as a key factor shaping intestinal development during fetal and neonatal stages. This review synthesizes recent studies on maternal nutrient intake—encompassing protein, energy, carbohydrates, minerals, vitamins, probiotics, and prebiotics—and their effects on intestinal growth and health of offspring. Emerging multi-omics evidence has revealed that gestational and lactational nutrition dynamically coordinates offspring intestinal development through vertical microbial transmission and epigenetic mechanisms, such as DNA methylation and histone acetylation. These processes further regulate intestinal barrier maturation, mucosal immunity, and enteroendocrine signaling. Collectively, this review emphasizes that enhancing maternal nutrition can promote postnatal growth by enhancing intestinal development and early microbial colonization in piglets. Further research is crucial to determining the optimal nutritional strategies during the perinatal period.

## 1. Introduction

The gastrointestinal tract (GIT) is widely recognized for its central role in digestion and absorption, and it constitutes one of the largest immunological organs in the body [1,2]. Additionally, the GIT functions as a critical barrier between the internal body and the external environment [3]. In pig production, intestinal health in piglets is a significant contributor to economic losses. Weaning is considered a pivotal event in intestinal development, often associated with severe enteric infections and multiple stress factors, including nutritional and environmental stressors [4,5]. Consequently, nutritional strategies for mitigating the adverse effects of weaning stress have become a major focus in the pig industry. In recent decades, extensive research has been conducted on intestinal development and health in weaning pigs, particularly in response to nutritional, psychological, and environmental stressors [4]. However, it is crucial to recognize that intestinal development initiates during the first trimester of gestation, underscoring the importance of fetal intestinal development in utero [6].

Adequate maternal nutrient intake during gestation is essential for fetal development and offspring health [7]. Following porcine embryo implantation, fetal growth and development depend on maternal nutrition and the intrauterine environment, which play critical roles in fetal programming and contribute to the differentiation, growth, and development of organs and tissues [8]. Fetal GIT growth is influenced by maternal diet, placental blood supply, and the extent of amniotic fluid swallowing [9]. Thus, nutrition has the potential to modulate GIT growth at various developmental stages during gestation. A growing body of evidence indicates that maternal diet during gestation exerts long-term effects on offspring intestinal development and microbiota composition [10]. Recent studies have demonstrated that enhancing maternal GIT microbiota composition and function can positively influence the development and maturation of the neonatal GIT [11]. A healthier maternal microbiota is likely to transfer beneficial microbes to the neonatal GIT, either through direct colonization or indirect effects on the succession of indigenous intestinal bacteria [12]. Furthermore, a growing a body of evidence is challenging the traditional assumption of a sterile intrauterine environment, suggesting that maternal–fetal microbial transmission occurs during gestation [13].

## 2. Intestinal Development (From Fetus to Neonates)

**Figure 1:**
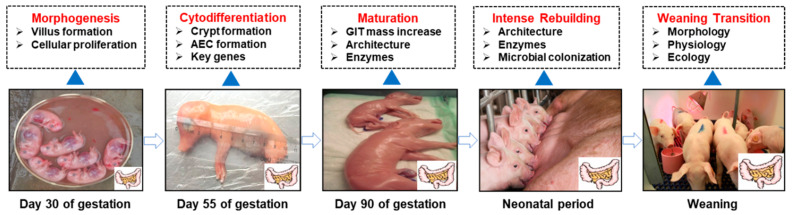
Onset of key development events in the gastrointestinal tract. AEC, apical endocytic complex [6,15].

Intestinal development is classically categorized into three distinct phases: the prenatal phase, marked by limited luminal stimulation; the neonatal phase (up to 72 h of after birth), which is a transition from the intrauterine to external environment and dependence on colostrum for passive immunity; the suckling phase, associated with milk suckling; and the weaning phase, characterized by adaptation to solid feed [3]. This progression is critical for ensuring adequate nutrient provision to the fetus and neonate. Given the structural and physiological similarities of GIT between pigs and human beings, pigs have been recognized as a valuable model for the investigation of clinical nutrition [6]. Figure 1 illustrates the intestinal development in relation to gestation, birth, and weaning in pigs.

The intestine of the fetal pig initiates development early in gestation, undergoing extensive morphologic and functional changes throughout this period [14]. The process encompasses mucosal morphogenesis, transitioning from a rudimentary tubular structure to one lined with villi, as well as the differentiation of immature epithelium into four major epithelial cell types [15]. By approximately day 40 of gestation, intestinal morphogenesis has progressed to the point where recognizable villi are present, and mRNA for enzymes and cytoskeletal proteins becomes readily detectable [16]. Between day 45 and 110 of gestation, the mass of GIT escalates over 170-fold in a cubic manner, increasing from 2.4% to 6.3% of total body mass. The most rapid GIT mass accretion occurs during the final gestational week despite minimal somatic growth [17]. Notably, during the last trimester of gestation, the increase in mucosal surface area is particularly dramatic, characterized by significant growth of both villi and microvilli. In the weeks preceding parturition, the pig intestine grows more rapidly than does the body overall, with its relative weight increasing by 70–80% in the last three weeks of gestation [18]. During this rapid growth period, the GIT is sensitive to luminal and systemic nutrients both before and after birth. Intestinal growth, along with increasing activity of brush border membrane enzymes and transporters, determines the intestinal capacity for the final stages of hydrolysis and absorption of dietary ingredients [19]. Furthermore, the development of porcine digestive enzymes closely resembles that of human development during the fetal and neonatal periods [6]. The fetal intestinal mass and the activities of brush-border enzymes increase significantly from days 70 to 110 of gestation [17].

After birth, the shift from parenteral to enteral nutrition induces marked architectural, ultrastructural, and functional intestinal remodeling [20]. Notably, neonatal pigs exhibit a 58% increase in total intestinal weight and an 80% surge in mucosal weight during the first 6 h of suckling, accompanied by a 4.6-fold elevation in mucosal DNA content by 24 h postpartum [21]. During the transition to weaning, pigs undergo nutritional, environmental, and psychological stresses, which are accompanied by significant changes in the structure and function of the small intestine. This change includes a reduction in villus height, an increase in crypt depth, a decrease in disaccharidase activity, and alterations in microbiota composition [5,22].

## 3. Immune System and Microorganism Colonization (From Fetus to Neonates)

Immune system ontogeny initiates during gestation and undergoes progressive maturation postnatally. Peripartum nutritional inputs critically influence neonatal immune system development, as fetal immunological programming exerts lasting effects on innate and adaptive immune competence [23]. Gut microbiota serves as a fundamental regulator of immune system activation and protective immunoglobulin induction [24]. Intestinal immune development commences prenatally, achieving functional maturation through postnatal microbial exposure. The sterile GIT of fetuses is rapidly colonized within 12 h after birth, with bacteria detectable throughout the entire GIT [25].

The microbiota present in early life is a significant determinant of immune and metabolic development, with potential long-lasting consequences. During the neonatal phase, microbial colonization drives mucosal barrier maturation and immune functionality in neonates [26]. Intestinal microbiota exhibits marked interindividual variation, modulated by environmental factors including dietary inputs [27]. Studies with germ-free piglets clearly demonstrates that bacteria are vital for the growth and development of the digestive tract [28]. Bacterial communities regulate transcriptional programs governing epithelial turnover and mucin biosynthesis while concurrently shaping mucosal immune system maturation and anti-inflammatory homeostasis [29]. The maternal gut microbiota during pregnancy and breastfeeding plays a critical role in shaping the gut microbiota of the offspring. Typically, maternal microbiota is considered the primary source for establishing the microbial community in newborns. Emerging evidence suggests microbial modulation represents a potential mechanism of developmental programming, whereby maternal dietary patterns may modulate offspring immune trajectories through microbiota-mediated pathways.

## 4. The Role of Maternal Nutrition in Intestinal Development and Microorganisms

The GIT development is orchestrated through the transition from placental nutrition prenatally to solely enteral nutrition postnatally, with maternal nutritional status emerging as a principal determinant of fetal intestinal development [30].

### 4.1. Protein and Amino Acids

Dietary proteins and amino acids (AAs) exhibit pleiotropic physiological functions extending beyond canonical roles in protein synthesis, particularly in intestinal homeostasis [31]. During gestation, an adequate supply of AAs critically regulates fetal growth trajectories, contingent on placental AA transport efficacy [32,33]. Maternal protein intake modulates intrauterine growth restriction (IUGR) incidence, small intestinal ontogeny, and systemic inflammatory signaling in offspring [34]. Compromised intestinal maturation may mediate postnatal compensatory growth mechanisms. Tuchscherer et al. (2012) demonstrated that maternal dietary protein–carbohydrate ratios during gestation influence neonatal humoral immunity and adaptive stress responses at weaning, as evidenced by altered cytokine profiles following acute immunogenic challenges in pig offspring [35]. The progeny of protein-restricted dams exhibit higher intestinal cell proliferation and upregulated transcriptional expression of sodium-glucose co-transporter 1 (SGLT1) and peptide transporter 1 (PEPT1) in the duodenum, with persistent immunoreactivity of SGLT1, glucose transporter 2 (GLUT2), and PEPT1 in adulthood mediated by transcriptional and proliferative adaptations [36]. Further, maternal protein restriction induces gastrointestinal dysfunction and enteric nervous system remodeling in rodent offspring, characterized by increased proportion of choline acetyltransferase immunoreactive neurons and suppressed autophagy in colonic myenteric neurons [37] (Table 1).

The intestinal microbiota plays a crucial role in the proliferation, apoptosis, immune response, and homeostasis of epithelial cells. The composition and diversity of intestinal microbiota are modulated by dietary protein profiles, although the effects exhibit species-specific patterns. A study in rats revealed that gestational high-protein diets reduce offspring fecal microbial diversity while enhancing milk microbiota diversity and cecal microbial richness, concurrently correlating with elevated abundance of putative probiotic taxa such as *Lactobacillus* spp. [38]. Contrastingly, maternal low-protein regimens in Huzhu Bamei suckling pigs enhanced microbial alpha diversity and reduced diarrheal incidence [39]. The differences of species and protein levels may explain these divergent outcomes. Furthermore, lactating sows supplemented with 1% spray-dried plasma and their offspring exhibited increased proportions of the phylum Bacteroidota and genus *Lactobacillus* and *Ruminococcus*, while displaying reduced levels of phylum Bacillota and genus *Bacteroides*, *Escherichia*/*Shigella*, and *Clostridium* [40]. Collectively, these findings implicate gestational microbial programming as a determinant of offspring intestinal development.

Essential AAs, acquired exclusively through dietary sources, function as fundamental constituents of polypeptides while orchestrating critical metabolic pathways governing cellular homeostasis, developmental processes, reproductive physiology, and immunoregulatory networks [41]. Sulfur AAs, particularly methionine (Met) and cysteine (Cys), exert indispensable roles in gastrointestinal physiology by facilitating luminal nutrient processing, sustaining mucosal immune functions, enhancing antioxidant ability, and reinforcing epithelial barrier against foreign antigens [42,43]. Perinatal optimization of maternal Met/Cys ratio enhances intestinal development and redox homeostasis in offspring piglets via synergistic mechanisms involving plasma metabolite profile alterations and microbiota compositional shifts. However, supra-nutritional Met exposure elevates susceptibility to metabolic dysregulation, emphasizing the necessity for precision in perinatal essential AA supplementation [44,45].

Maternal non-essential AAs also play an important role in the intestinal development of offspring. Wang et al. (2020) has demonstrated that maternal supplementation with 1% proline (Pro) enhances fetal growth and enterocyte proliferation through polyamine biosynthesis [46]. L-arginine is integral to multiple pathways critical for piglet growth and development, including nitric oxide synthesis, energy metabolism, polyamine synthesis, cellular protein production, muscle accretion, and the synthesis of other functional amino acids [14]. A recent study has demonstrated that maternal L-arginine supplementation could improve the live litter weight at birth and litter weight gain during lactation, but there is no substantial data to support the intestinal development in offspring piglets [47]. Glutamine (Gln) serves not only as an energy substrate and precursor for protein biosynthesis but also as a signaling molecule regulating intestinal gene expression, cell proliferation, immune function, and redox homeostasis [48]. Maternal dietary Gln supplementation has been proposed as a strategy to enhance fetal growth and mitigate IUGR occurrence. Zhu et al. (2018) reported that supplementation in sows with 1% Gln from day 85 of gestation until farrowing alleviated IUGR-induced intestinal impairment by suppressing intestinal miR-29a levels and increasing extracellular matrix and tight junction protein abundance [49]. Overall, beyond essential AAs, particular attention should be given to highlighting the critical role of maternal non-essential AAs in offspring’s intestinal development (Table 1). 

**Table 1:** Effects of maternal protein and amino acid intake on reproductive performance of sows and the intestinal development of the offspring.

Sources	Experimental Groups	Stage	Findings	Study
Pro	(1) CON (2) 1% Pro (3) 1% Pro + 0.0167% adifluoromethylornithine	From day 15 to 70 of gestation	Fetal weight (+) Protein and DNA concentrations of the fetal small intestine (+) mRNA levels for potassium voltage-gated channel, shaker-related subfamily, and member 1 (Kv1.1) in the fetal small and large intestines (+)	[46]
Arg	(1) CON (2) CON+ 0.28% Arg (3) CON+ 0.79% Arg	Day 70 of gestation to farrowing	Live litter weight (+) Piglet weight gain and litter weight gain (+)	[47]
Met and Cys	Met/Cys: 46% Met, 51% Met, 56% Met, and 62% Met	Day 90 of gestation to lactation	46% Met: hippuric acid, retinoic acid, riboflavin, and δ-tocopherol (−) 51% Met: Firmicutes (+) (the optimum Met/Cys ratio) 62% Met: Proteobacteria (+)	[44]
Protein	(1) Normal protein: 14% CP (2) Low protein: 12% CP (3) Very low protein: 10%	During gestation	Low protein: the abundance and diversities of the jejunum microbiome (+); biological functions of the jejunum microbiome (+)	[39]
Glutamine	(1) CON (2) CON + 1% Gln	Day 85 of gestation to farrowing	Average birth weight (+) Gln concentration (+) Intestinal weight and morphologies (+) Extracellular matrix, tight junction protein (+)	[49]
Met	(1) CON (2) CON + 0.12% Met (3) CON+ 0.24% Met	Day 90 of gestation to day 21 of lactation	Malondialdehyde (−) glutathione peroxidase (+) Phascolarctobacterium and Bacteroidetes (+)	[45]
Met	(1) Met (2) HMTBA	During lactation	Reduced glutathione (+) Oxidized glutathione (GSSG)/GSH ratio (−) Glutathione peroxidase (+) Villus height and goblet cell numbers (+)	[50]
Protein	(1) Adequate protein (AP, 12.1%) (2) Low protein (LP, 6.5%) (3) High protein (HP, 30%)	During gestation	LP: Mortality (+) Cortisol (+) IL-6 (+) HP: CD4+ cell percentage (+) CD4+/CD8+ ratio (+) IL-6 (+)	[35]
Protein	(1) Adequate protein (AP, 12.1%) (2) Low protein (LP, 6.5%) (3) High protein (HP, 30%)	During gestation	HP and LP: body weight and catch-up growth (−) Ki67 and active caspase 3 (−) IUGR: brush border enzyme activities (−) Vacuolated enterocytes disappearance (−)	[34]
Protein	(1) CON (2) CON + 1% SDP	Day 30 before farrowing to weaning	Phylum Bacteroidota and genus *Lactobacillus* and *Ruminococcus* (+) Phylum Bacillota and genus *Bacteroides*, *Escherichia/Shigella*, and *Clostridium* (−)	[40]

+, increase; −, decrease; CON, control group.

### 4.2. Fatty Acids

Fatty acids serve as a major energy source, structural components of cell membranes, metabolic substrates in biochemical pathways, signaling molecules, and immunomodulators [51]. Medium-chain triglycerides (MCT), long-chain polyunsaturated fatty acid (LC-PUFA), and SCFA are indispensable for neonatal energy metabolism and GIT development [31]. In our pervious study, maternal over-nutrition (150% of National Research Council [NRC] recommendations) enhanced intestinal function in newborn and weaned piglets by upregulating digestive enzyme activity and transporter gene expression, whereas maternal undernutrition (75% of NRC recommendations) impaired fetal intestinal development due to intrauterine nutrient restriction [52] (Table 2). Maternal energy intake during gestation significantly influences fetal and postnatal outcomes, including birth weight, small intestinal weight, and intestinal morphology, which correlate with changes in intestinal digestion and absorption capacity [53,54]. Reduced maternal energy intake may also elevate intestinal inflammation risk by compromising epithelial barrier integrity [54]. In studies involving primates and humans, maternal high-fat diets during gestation have been shown to reduce offspring intestinal microbiota diversity and persistently alter gut microbial composition [55,56].

n-3 PUFA, particularly eicosapentaenoic acid (EPA) and docosahexaenoic acid (DHA), are essential nutrients that must be obtained exogenously. Inadequate dietary intake of these fatty acids elevates the risk of maternal undernutrition and fetal growth impairment. Innis et al. (2010) demonstrated that maternal dietary supplementation with n-3 LC-PUFA during gestation and lactation augmented colonic permeability and exacerbated inflammatory responses to chemically induced colitis in offspring rats [57]. Parallel findings by Boudry and colleagues revealed that gestational and lactational diets enriched with α-linolenic acid (18:3 n-3) elevated jejunal permeability in piglets [58]; furthermore, such supplementation increased intestinal lipopolysaccharide (LPS) translocation and promoted an anti-inflammatory immune profile in neonatal piglets upon LPS exposure [59]. The intestinal epithelial barrier is critical for maintaining gut homeostasis and shaping neonatal immune system development. Diet-induced neuroplastic modifications in the enteric nervous system (ENS), a primary regulator of gastrointestinal function, may underlie alterations in intestinal permeability. Piglets from sows receiving linseed-oil-supplemented diets during gestation and lactation exhibited transient increases in ileal permeability, likely mediated by ENS architectural and functional neuroplastic adaptations, alongside diminished ileal barrier reactivity to mast cell degranulation [60]. Intestinal health is closely linked to intestinal microbiota composition, with emerging evidence underscoring the substantial influence of dietary fish oil or n-3 PUFA on microbial communities. Maternal fish oil supplementation (60 g/d) during late gestation and lactation enhances growth performance, improves immune function, and reduces post-weaning diarrhea incidence in piglets. These benefits correlate with elevated α-diversity of fecal microbiota and increased abundance of *Lactobacillus* species [61]. Further, glucose is a major energy source for weaning piglets. Gestational n-3 PUFA-rich diets enhance intestinal glucose absorption and elevate muscle glycogen reserves in newly weaned pigs, which are associated with upregulated intestinal mRNA expression of GLUT2 and SGLT-1 [62]. In summary, maternal dietary fatty acid interventions can modulate gastrointestinal health and function in offspring pigs, enhancing immune homeostasis and disease resistance during the post-weaning period.

**Table 2:** Effects of maternal energy intake on reproductive performance of sows and offspring’s intestinal development.

Source	Experimental Groups	Stage	Key Findings	Study
Fat sources	(1) Lard (2) Linseed oil	During gestation and lactation	18:3(n-3) and 20:5(n-3) in maternal RBC and piglet ileum (+) 22:6(n-3) and 20:4(n-6) in maternal RBC and piglet ileum (−) 18:3(n-3) in milk and piglet ileum (+)	[60]
Energy level	(1) NRC, 2012 (3.40 MCal DE) (2) Low-energy diet (3.00 MCal DE)	Day 1 of gestation to farrowing	Small intestinal weight (−) The ratio of villus height to crypt depth (−) Lactase and sucrase (−) IL-6, TNF-a (+) TLR-4, IL-1b and NF-kB (+) ZO-1 (−)	[54]
Energy level	(1) NRC, 2012 (2) High-energy diet (add 4.6% soybean oil)	Day 1 of gestation to farrowing	Small intestinal weight (+) Villus height (+) Lactase, sucrase (+) Insulin-like growth factor 1 receptor (+)	[53]
Fat sources	(1) 18:3n-3 (2) 18:2n-6	Throughout gestation and lactation	Mesenteric lymph nodes (+) MHC class II+ antigen-presenting cells (+)	[59]
Nutrition level	(1) 75% of NRC (2) NRC (3) 150% of NRC	Day 1 of gestation to farrowing	Small intestine weight (+) Brush-border lactase (+) SGLT1, GLUT2, PEPT1, and GLP2R (+)	[52]
Fat sources	(1) Lard (2) Linseed oil	During gestation and lactation	Permeability (+) Paracellular permeability (−) Choline acetyltransferase (ChAT)-immunoreactive (IR) neurons (+)	[58]
Fat sources	(1) CON (2) CON + fish oil (3) CON + gold fat (4) CON + coconut fat	Entire gestation and lactation periods	(2) (3): Glucose transporter 2 (+) Sodium glucose transporter 1 protein (+) AMP-activated protein kinase activity (+)	[62]
Fish oil	(1) CON (2) CON + 30 g/d fish oil (3) CON + 60 g/d fish oil	Day 90 of gestation to weaning at day 21 of lactation	Plasma IgG, IgM and IgA (+) Cortisol (−) α-diversity of fecal microbiota, *Lactobacillus* genus (+)	[61]

+, increase; −, decrease; CON, control group.

### 4.3. Carbohydrate

Carbohydrates constitute a critical nutritional component in swine feed formulations, representing 60–70% of total dietary content. These compounds vary structurally from simple sugars (e.g., monosaccharides and disaccharides) to complex polymers, including starch and nonstarch polysaccharides—the latter categorized collectively as dietary fiber [63]. Indigestible carbohydrates serve as the primary energy substrate for gut microbiota, which metabolize them into short-chain fatty acids (SCFAs) through anaerobic fermentation. The fermentability and physicochemical properties of dietary carbohydrates directly modulate microbial community composition and diversity. For instance, Metzler-Zebeli et al. (2010) demonstrated that altering carbohydrate fermentability and viscosity in swine diets reshapes intestinal microbial diversity [64].

Emerging evidence indicates that maternal environmental and phenotypic factors significantly influence offspring phenotypic traits and biological fitness. Strategic modulation of sow microbiota through dietary fiber interventions may directly shape offspring intestinal microbial communities [65]. In our recent study, gestational high-fiber intake elevated proteins associated with oxidative homeostasis, energy metabolism, and immune-inflammatory responses in neonatal piglets while reducing apoptosis-related proteins and those governing cytoskeletal organization and cellular motility in the small intestine. These molecular shifts coincided with structural adaptations in intestinal morphology and microbial community restructuring [66]. Gut microbiota composition demonstrates marked sensitivity to the physicochemical properties of dietary fibers. Gestational fiber supplementation alters sow gut microbiota diversity and composition, systemically influencing nutritional metabolism, physiological adaptation, and immune regulation. For instance, maternal wheat bran and inulin administration during gestation and lactation modifies offspring microbial fermentation patterns [67], with inulin further suppressing *Enterobacteriaceae* populations [68] (Table 3). Cheng et al. (2018) demonstrated that gestational soluble fiber supplementation (2.0% pregelatinized waxy maize starch and guar gum) enhances offspring growth rates and reduces diarrhea incidence, correlating with improved intestinal barrier integrity (evidenced by plasma zonulin, endotoxin, and diamine oxidase levels) and microbiota-mediated metabolic reprogramming, including increased *Bacteroides*, *Lactobacillus*, *Roseburia*, *Fusobacterium*, and *Acinetobacter* abundance [10]. Beyond merely increasing fiber levels, the source of dietary fiber is equally critical. Liu et al. (2021) demonstrated that adding diverse fiber sources (alfalfa meal, beet pulp, and soybean skin) into maternal diets during mid-to-late gestation modulated sow and offspring performance [69]. Notably, alfalfa meal supplementation markedly enhanced maternal and neonatal performance while alleviating gastrointestinal and systemic inflammatory responses. Additionally, this intervention significantly elevated the relative abundance of anti-inflammatory bacterial taxa and reduced proinflammatory microbial populations [69]. Collectively, these findings underscore maternal nutritional programming as a critical determinant of offspring microbial–immune axis development, warranting further investigation into targeted dietary fiber. 

**Table 3:** Effects of maternal carbohydrate intake on reproductive performance of sows and offspring’s intestinal development.

Source	Experimental Groups	Stage	Key Findings	Study
Soluble fiber	(1) CON (2) CON + 2.0% pregelatinized waxy maize starch plus guar gum (SF)	During gestation	Growth rate (+) The incidence of diarrhea (−) The fecal and plasma levels of acetate and butyrate (+) Plasma zonulin and fecal lipocalin-2 (−) Plasma concentrations of interleukin 10 (IL-10) and Transforming growth factor (TGF-β) (+) *Lactobacillus* spp. (+) *Bilophila* spp. (−)	[10]
RS	(1) Containing 33% of digestible starch (DS diet) (2) Containing 33% of pea starch (RS) diet	Gestation and lactation	Firmicutes/Bacteroidetes ratio (+) Bifidobacterium (+) Milk protein concentration (−) Lactose concentration (+) Zonula occludens 1 (ZO-1) (+)	[70]
Fiber	Insoluble/soluble fiber ratio of 3.89 (R1), 5.59 (R2), 9.12 (R3), and 12.81 (R4)	During the entire gestation	Duodenal weight, jejunal villus height, and villus height/crypt depth (−) Lactase, sucrase, and maltase (−) Antioxidant capacity (−) Inflammatory response (+)	[71,72]
Fiber	(1) Control diet (CD, 16.15% dietary fiber) (2) High-dietary-fiber diet (HFD, 30.14% dietary fiber)	Day 90 of gestation to farrowing	α-diversity indices (+) Acidobacteria and Bacteroidetes at phylum level (+) *Bradyrhizobium* and *Phyllobacterium* at genus level (+) The abundances of proteins associated with oxidative status, energy metabolism, and immune and inflammatory responses (+)	[66]
Fiber	(1) CON (2) CON + sugar beet pulp (SBP) (3) CON + wheat bran (WB)	Day 85 of gestation to weaning	SBP: sow ADFI, litter. and piglet weaning weight, piglet ADG, immunoglobulin A (IgA), and interleukin-10 (IL-10) levels in the colostrum and IgA levels in the milk (+) Christensenellaceae and butyrate levels in the colon (+) WB: IL-10 levels in the milk (+) *Lactobacillaceae* in the colon (+)	[73]
RS	Maternal diets: digestible starch (DS) or RS diet Piglet treatment: control diet or high fat diet	Late gestation and lactation	RNA sequencing on liver and colon scrapings revealed minor differences	[74]
Inulin	(1) CON (2) CON + 3% inulin	Gestation and lactation	The cell numbers of *enterococci* (+) Cell numbers of *eubacteria* (stomach) and *C. leptum* (caecum) (+) Cell numbers of *enterobacteria* and *L. amylovorus* (stomach) (−)	[68]
Fiber source	(1) CON (2) Alfalfa meal (3) Beet pulp (4) Soybean skin	Day 60 of gestation to farrowing	Alfalfa meal improved sow and piglet performance and relieved gut and systemic inflammation	[69]

+, increase; −, decrease; CON, control group.

### 4.4. Minerals and Vitamins

Research on the effects of maternal mineral and vitamin on the intestinal health of offspring remains limited. Zinc (Zn) is an essential micronutrient, serving as a cofactor or structural component for numerous enzymatic and metalloenzymatic systems while also regulating acid–base homeostasis and osteochondral development. Payne et al. (2006) observed that offspring from sows receiving ZnSO_4_ supplementation exhibited greater duodenal and ileal villus width compared to ZnAA groups, implying that maternal Zn form influences offspring intestinal morphogenesis [75] (Table 4). Selenium (Se) attenuates intestinal inflammation by reducing oxidative stress. A comparative analysis of maternal sodium selenite (Na_2_SeO_3_) and 2-hydroxy-4-methylselenobutanoic acid (HMSeBA) supplementation demonstrated that gestational HMSeBA administration enhances intestinal antioxidant capacity and suppresses inflammatory signaling (e.g., NF-κB and ERK/Beclin-1 pathways) in offspring pigs [76]. Similarly, compared with Na_2_SeO_3_, maternal selenium-enriched yeast (SeY) supplementation during late gestation and lactation improves piglet growth performance, selenium status, antioxidant capacity, and immunoglobulin transfer during the first week of lactation. This supplementation also modulates fecal microbiota in sows by enriching antioxidant-associated and SCFA-producing microbiota. These adaptations enhance small intestinal barrier function and activate the Nrf2/Keap1 pathway in offspring [77].

A mouse study indicated that maternal vitamin D deficiency elevates intestinal permeability in offspring, potentially mediated by the epigenetic suppression of the Wnt/β-catenin signaling pathway via histone modifications [78]. In addition, vitamins and their derivatives are critical regulators of intestinal development. Maternal 25-hydroxycholecalciferol (25-OH-D_3_) administration enhances dietary calcium bioavailability, modifies lactational milk composition and fatty-acid profiles, and elevates hindgut butyrate concentrations in suckling piglets through the upregulation of calcitropic genes [79,80]. Vitamin A (VA) exerts pleiotropic effects on immune function, playing a critical role in mucosal immunity and intestinal lymphocyte trafficking. Supplementation with 30,000 IU VA during the third trimester in porcine epidemic-diarrhea-virus-(PEDV)-infected gilts demonstrated dual therapeutic benefits for both maternal and neonatal health. VA-supplemented gilts exhibited elevated anti-PEDV IgA levels in systemic circulation, mammary secretions, and ileal tissue, correlating with improved survival rates in PEDV-challenged neonates from VA-treated dams [81].

**Table 4:** Effects of maternal minerals or vitamins intake on the reproductive performance of sows and offspring’s intestinal development.

Source	Experimental groups	Stage	Key Findings	Study
Mineral	(1) CON (contains 100 ppm Zn from ZnSO_4_) (2) CON + 100 ppm Zn from ZnSO_4_ (3) CON + 100 ppm additional Zn from ZnAA	Day 15 of gestation and continuing through lactation	ZnSO_4_: duodenal villus width (+) ZnAA: ileal villus width (−)	[75]
Mineral	(1) Na_2_SeO_3_ (2) Selenium-enriched yeast	Day 85 of gestation and continuing through lactation	Se content in the plasma and milk (+) T-AOC and GSH-Px in the colostrum (+) Protein abundances of MUC1, E-cadherin, ZO-1, occludin, and claudin (+) SCFA-producing microbiota (+)	[77]
Mineral	(1) Na_2_SeO_3_ (2) HMSeBA	During gestation	Ileal GPX2 and SePP1 (+) IL-1β, IL-6 and NF-κB genes (−) p-NF-κB, Beclin-1 and p-ERK proteins (−)	[82]
Vitamin	(1) 2000 IU/kg vitamin D_3_ (2) 50 μg/kg 25-OH-D_3_	Day 107 of gestation to day 21 of lactation	Milk n-6/n-3 PUFA ratio (+) Bone-specific alkaline phosphatase (+) Calcium absorption rate (+) Milk fat content and immunoglobulin G level (+) Concentration of butyrate (+)	[79,80]
Vitamin	(1) Mock (2) Mock + Vitamin A (VA) (3) PEDV (4) PEDV + VA	Day 76 of gestation throughout lactation	Maternal IgA (+) Lactogenic immune protection in nursing piglets (+)	[81]

+, increase; −, decrease; CON, control group; 25-OH-D_3_, 25-hydroxycholecalciferol.

### 4.5. Probiotics and Prebiotics

Emerging evidence indicates that maternal administration of probiotics and prebiotics modulates GIT microbiota composition and functionality, thereby beneficially influencing offspring GIT development and maturation [83]. Gestational and lactational probiotic supplementation in sows alters intestinal microbiota profiles and mucosal cytokine levels in suckling piglets [84]. Maternal microbiota transmission to offspring occurs via parturition-associated contact or lactation-derived breast milk, shaping neonatal gut microbial colonization patterns [85]. Early microbial colonization critically stimulates mucosal immune system development and functional maturation [86]. Probiotics are defined as live microorganisms administered in sufficient quantities to confer GIT microbial equilibrium [87]. Maternal dietary supplementation with *Bacillus subtilis* C-3102 during gestation-lactation phases modifies fecal microbiota in sows and post-weaning piglets [88]. Synergistic mixed probiotic formulations enhance pathogen resistance, intestinal health, and nutrient utilization. For instance, maternal supplementation with a multi-strain probiotic (*Lactobacillus helveticus* BGRA43, *Lactobacillus fermentum* BGHI14, *Streptococcus thermophilus* BGVLJ1-44) elevates neonatal piglet microbiota diversity [89]. Wang et al. (2020) demonstrated that gestational–lactational synbiotic supplementation (compound probiotics [*L. Plantarum B90* and *S. cerevisiae P11*] with xylo-oligosaccharide) increases beneficial bacteria (*Bifidobacterium* and *Lactobacillus*), suppresses pathogenic *Escherichia coli*, enhances colonic SCFAs absorption, and improves the immunity and antioxidant capacity in offspring piglets [90]. In addition, probiotic supplementation also shortens farrowing duration and enhances suckling piglet growth performance via persistent gestational–lactational modulation of intestinal microbiota [91,92]. Delivery and weaning are significant stressors for both sows and piglets, negatively impacting reproductive and growth performance. Numerous studies have demonstrated that diets supplemented with probiotics can improve sow reproductive performance and the viability of their progeny [93,94]. Recent studies indicate that probiotics can regulate sow milk metabolism through the gut–mammary axis, influencing the structure of the offspring’s gut microbiota and improving their immune status and antioxidant capabilities [95] (Table 5). However, some reports reported no improvement in the productive performance of sows or piglet growth due to the administration of duration time and dose [96].

Beyond probiotics, prebiotics serve as natural feed additives by functioning as selectively metabolized substrates that promote beneficial microbial activity and host health [97]. Defined as non-digestible carbohydrates and oligosaccharides, prebiotics occur naturally in human and animal diets, with structural diversity influencing their functional specificity [98]. Maternal supplementation with 10.0 g/day seaweed extract (SWE; containing laminarin [1.0 g], fucoidan [0.8 g], and ash [8.2 g]) exhibits significant immunomodulatory effects, evidenced by elevated colostral IgA and IgG concentrations and upregulated ileal TNF-α mRNA expression following ex-vivo LPS challenge, as well as reduces sow fecal *Enterobacteriaceae* abundance at parturition and suppresses *Escherichia coli* colonization in piglets at weaning [99,100]. Sow-derived milk oligosaccharides serve as fermentable substrates for neonatal microbiota, suggesting their pivotal role in early microbial colonization patterns [67,101]. Lactational supplementation with short-chain fructooligosaccharides (scFOS) enhances microbial fermentative capacity in suckling piglets, stimulating intestinal immune maturation through elevated ileal cytokine production, expanded goblet cell populations, and enhanced vaccine-specific IgA responses [102]. This enhanced fermentative capacity may stem from scFOS-induced modifications in milk oligosaccharide profiles, consistent with findings indicating that lactating sows supplemented with chitooligosaccharide (COS) have a significantly increased milk oligosaccharide composition [103]. scFOS-supplemented sows exhibit elevated colostral IgA and TGF-β1 concentrations, potentiating neonatal mucosal immunity through enhanced secretory IgA synthesis in Peyer’s patches and T-cell activation [104]. Duan et al. (2019) observed that maternal and/or offspring mannan-oligosaccharide (MOS) supplementation improves intestinal microbial profiles, strengthens mucosal immunity, and attenuates systemic inflammation in piglets [105]. Indigestible oligosaccharides and polysaccharides represent a safer alternative to probiotics for maternal GIT microbiota modulation, thereby benefiting neonatal health [106].

In addition to the prebiotics discussed above, some nutraceuticals and functional nutrients have demonstrated utility in pig industry. Maternal L-carnitine supplementation during gestation and lactation enhances intestinal barrier integrity in both neonatal and 21-day post-weaning piglets [107]. Gestational resveratrol supplementation mitigates weaning-associated diarrhea and intestinal inflammation while improving offspring intestinal morphology, likely through microbiota modulation and transcriptional regulation [108]. Furthermore, dietary mannan-rich fractions during gestation–lactation cycles elevate colostral and milk IgG levels, critical for neonatal intestinal and immune development, while inducing gene expression patterns suggestive of long-term performance enhancements [109]. Recent studies have identified spatially distinct microbial colonization trajectories across intestinal mucosa, driven by vertically transmitted maternal milk and gut microbiota, which prime neonatal immune and barrier function establishment [110]. Overall, the supplementation of diet with probiotics and prebiotics for gestating and lactating sows accelerates offspring small intestinal epithelial maturation and is coupled with enhanced mucosal immunity and colostral antibody transfer during early postnatal stages [111].

**Table 5:** Effects of maternal probiotic or prebiotic supplementation on the reproductive performance of sows and offspring’s intestinal development.

Source	Experimental Groups	Stage	Key Findings	Study
Probiotic mixture	(1) CON (2) CON+ probiotic mixture	Day 0 of gestation until 20 days before delivery	Litter size and litter weigh at birth (+) Diarrhea incidence (−) Antioxidant capabilities Systemic immune status	[95]
Seaweed extracts (SWE)	2 × 2 factorial design (1) CON (2) CON + SWE 10.0 g/d LPS challenge	From day 107 of gestation until weaning (d 26)	Colostrum IgA, IgG, and serum IgG (+) Colonic *E. coli* population (−) TNF-α mRNA expression (+)	[99]
Seaweed extracts (SWE)	2 × 2 factorial design - SWE vs. +SWE - ETEC vs. +ETEC	Day 83 of gestation until weaning (day 28)	Heat-labile enterotoxin gene (−) Villus height in the ileum (+)	[100]
Methyl donor (MET)	(1) CON (2) BPA (50 mg/kg) (3) MET (3 g/kg betaine, 400 mg/kg choline, 150 µg/kg vitamin B12, and 15 mg/kg folic acid) (4) BPA + MET	Throughout gestation	The ratio of villus height to crypt depth (+) Lactase activity (+) Pept1, DNMT1, DNMT3a, and MTHFR (+) DNA methylation level of jejunum Pept1 (+)	[112]
Prebiotics (scFOS)	(1) CON (2) Mycotoxin deoxynivalenol (3) scFOS	During the last 4 weeks of gestation	T regulatory response (+)	[113]
scFOS	(1) CON (2) CON + scFOS	During the last third of gestation and throughout lactation	Ileal cytokine secretions (IFN)-γ (+) Cecal goblet cell number (+) IgA vaccine response (+) Bacterial fermentative activity (+) Colonic butyrate (+)	[102]
Prebiotics	Received daily 45 mL lactulose	10 days before until 10 days after parturition	Daily weight gains (+) Total aerobic bacterial counts and *C. perfringens* counts (+) IgG antibody levels (+)	[114]
Resveratrol	(1) CON (2) CON + 300 mg/kg resveratrol	20 days after breeding through gestation and lactation	Butyrate-producing bacteria (+) Diarrhea and intestinal inflammation (−) Intestinal morphology (+) T-cell receptor, MAPK, and Ras signaling (−)	[108]
Mannan oligosaccharide (MOS)	2 × 2 factorial design Sow: (1) CON (2) CON + 400 mg/kg MOS Piglets: (1) CON (2) +800 mg/kg MOS	Day 86 of gestation until weaning	*Lactobacillus* (+) *Escherichia coli* (−) sIgA content (+) Toll-like receptor 2 (TLR2), toll-like receptor 4 (TLR4), and interleukin 8 (IL-8) (−) Cytokines IL-2 and IL-4 (−)	[105,115]
L-carnitine	2 × 2 factorial design (soyabean meal vs. DDGS) and two L-carnitine levels	Gestation and lactation	Total superoxide dismutase activity (+) Malondialdehyde (−) IL-1β, IL-12, IL-6, and TNF-α (−) *Lactobacillus* spp. and *bifidobacteria* spp. (+) Tight junction proteins (+)	[107]
Yeast mannan-rich fraction (MRF)	(1) CON (2) CON + MRF (900 mg/kg)	Gestation and lactation	Protein and immunoglobulin G (IgG) in milk (+) Genes related to tissue development, functioning. and immunity, as well as greater cell proliferation and less migration of cells (+)	[109]
Short-chain fructooligosaccharide (scFOS)	(1) CON (2) CON + (10 g scFOS/d)	The last 4 weeks of gestation and the 4 weeks of lactation	IgA, scFOS. and TGFb1 concentrations (+)	[104]
Oregano essential oils (OEO)	(1) CON (2) CON + 250 mg/kg of OEO	Gestation and lactation	Fat percentage in milk (−) T lymphocytes (+)	[116]
Bioactive substances	(1) CON (2) CON + flax seed, rapeseed, linden inflorescence, taurine, L-carnitine and tocopherol acetate	Day 80 of gestation to lactation	Apoptotic index (−) p53 expression (−)	[111]
Folic acid (FA)	(1) CON (1.8 mg FA per kg) (2) FA-supplemented diet (30.3 mg FA per kg)	During gestation	DNMT-1 and Bcl-2 (+) p53, Bax, Mpg, and Apex-1 (−)	[112]

+, increase; −, decrease; CON, control group; MTHFR, methylenetetrahydrofolate reductase.

## 5. The Underlying Mechanism of Maternal Nutrition and Offspring Development

Accumulating evidence suggests that the impact of early nutrition on chronic diseases in offspring may be mediated through epigenetic mechanisms [101]. Epigenetic processes, including DNA methylation, posttranslational histone modifications (e.g., acetylation, methylation, phosphorylation, ubiquitination), and microRNA abundance, are critical for the normal development of tissues across various life stages [117]. Maternal nutritional intake during gestation has been shown to influence the fetal and postnatal epigenome and transcriptome, resulting in measurable changes in intestinal growth [118]. In a recent study, alterations in differentially methylated regions (DMRs) were identified in the intestinal tissues of piglets with IUGR, with these DMR-associated genes interacting with key proteins involved in immunity and metabolism. These findings suggest that IUGR induces DNA methylation changes in intestinal tissues, potentially modulating the expression of genes associated with cell apoptosis, differentiation, and immune function [119]. As a pivotal epigenetic modification, DNA methylation plays a crucial role in transcriptomic regulation [120]. DNA methylation is affected by several external factors such as environmental exposures, nutrient intake, and disease occurrence [121]. Emerging evidence in nutritional epigenetics indicates that maternal supplementation with methyl donors (e.g., betaine, choline, folic acid, vitamin B12) can alter DNA methylation patterns, resulting in lasting phenotypic changes in offspring [122,123]. In our recent study, maternal dietary supplementation with a methyl donor mixture (3 g/kg betaine, 400 mg/kg choline, 150 µg/kg vitamin B12, and 15 mg/kg folic acid) mitigated bisphenol A-induced impairments in intestinal morphology, disaccharidase activity, and nutrient transporter gene expression in offspring piglets by enhancing DNA methylation [112]. Furthermore, maternal methyl donor supplementation exerts persistent effects on the colonic mucosa microbiota of offspring, implicating bacterial involvement in the epigenomic reprogramming of gut function [103]. Beyond DNA methylation, recent studies have demonstrated that maternal supplementation with mineral methionine hydroxy analog chelate can influence histone acetylation and fetal programming, potentially regulating intestinal health and skeletal muscle development in piglets at birth and weaning, thereby promoting enhanced growth [124]. Similarly, in a poultry study, a maternal high-zinc diet attenuated intestinal inflammation in offspring chicks by reducing DNA methylation and increasing H3K9 acetylation in the A20 promoter region [125]. These findings collectively demonstrate that epigenetic gene regulation can be modulated by nutritional interventions, leading to altered gene expression patterns [120]. In summary, maternal supplementation with enzyme cofactors and methyl donors may induce changes in DNA methylation and histone acetylation, thereby influencing global gene expression and biological responses in offspring [118,126].

## 6. Conclusions and Future Perspectives

**Figure 2:**
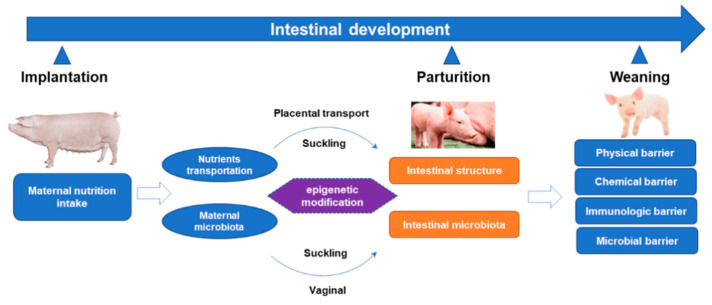
Summary of contributions of maternal nutrition to the intestinal development and composition of microbiota in offspring pigs.

Intestinal development in piglets is a complex, multifactorial process that plays a critical role in ensuring the health and productivity of piglets within production systems (Figure 2). The prenatal and early postnatal periods represent a crucial window for the development of the intestinal tract and immune system. Numerous studies have demonstrated that imbalances in macronutrients, vitamins, minerals, prebiotics, and probiotics—whether provided in excess or deficiency during pregnancy and/or lactation—can exert long-lasting, often detrimental effects on progeny. In this review, we mainly focus on the positive effects of maternal nutrition in the regulation of intestinal development of offspring piglets during gestation and lactation. Based on this review, it can be concluded that appropriately increasing fiber intake during late gestation can improve the phenotype of offspring by regulating intestinal microbiota and alleviating inflammation. Perinatal prebiotic and probiotic administration can modulate offspring health and intestinal development via the gut–mammary axis. Furthermore, given the rapid and profound changes in intestinal development during the perinatal period, further investigation is warranted to optimize dietary composition during this critical phase to support optimal intestinal development in offspring.

The intestinal microbiota plays a pivotal role in immunological development and metabolic health. Emerging evidence suggests that maternal microbiota influences offspring gut colonization through direct exposure during birth (via the vaginal canal) and through breast milk during lactation, thereby contributing to immune system development and long-term health outcomes in offspring (Figure 2). However, in our recent study, we observed distinct colonic microbiota compositions in newborn piglets (prior to suckling) derived from sows fed different fiber diets during gestation [66]. This finding challenges the traditional assumption of a sterile intrauterine environment, suggesting instead that maternal–fetal microbial transmission occurs during gestation. Consequently, further research is necessary to elucidate the mechanisms by which maternal nutrition during gestation influences offspring microbiota.

## Data Availability

No new data were created or analyzed in this study.
